# A genome-wide association study in the Japanese population identifies the 12q24 locus for habitual coffee consumption: The J-MICC Study

**DOI:** 10.1038/s41598-018-19914-w

**Published:** 2018-01-24

**Authors:** Hiroko Nakagawa-Senda, Tsuyoshi Hachiya, Atsushi Shimizu, Satoyo Hosono, Isao Oze, Miki Watanabe, Keitaro Matsuo, Hidemi Ito, Megumi Hara, Yuichiro Nishida, Kaori Endoh, Kiyonori Kuriki, Sakurako Katsuura-Kamano, Kokichi Arisawa, Yora Nindita, Rie Ibusuki, Sadao Suzuki, Akihiro Hosono, Haruo Mikami, Yohko Nakamura, Naoyuki Takashima, Yasuyuki Nakamura, Nagato Kuriyama, Etsuko Ozaki, Norihiro Furusyo, Hiroaki Ikezaki, Masahiro Nakatochi, Tae Sasakabe, Sayo Kawai, Rieko Okada, Asahi Hishida, Mariko Naito, Kenji Wakai, Yukihide Momozawa, Michiaki Kubo, Hideo Tanaka

**Affiliations:** 1 Division of Epidemiology and Prevention, Aichi Cancer Center Research Institute, Nagoya, Japan; 2 Department of Preventive Medicine, Nagoya University Graduate School of Medicine, Nagoya, Japan; 3 Division of Biomedical Information Analysis, Iwate Tohoku Medical Megabank Organization, Disaster Reconstruction Center, Iwate Medical University, Morioka, Japan; 4 Division of Molecular and Clinical Epidemiology, Aichi Cancer Center Research Institute, Nagoya, Japan; 5 Department of Epidemiology, Nagoya University Graduate School of Medicine, Nagoya, Japan; 6 Department of Preventive Medicine, Faculty of Medicine, Saga University, Saga, Japan; 7 Laboratory of Public Health, Division of Nutritional Sciences, School of Food and Nutritional Sciences, University of Shizuoka, Shizuoka, Japan; 8 Department of Preventive Medicine, Institute of Biomedical Sciences, Tokushima University Graduate School, Tokushima, Japan; 9 Department of International Island and Community Medicine, Kagoshima University Graduate School of Medical and Dental Sciences, Kagoshima, Japan; 10 Department of Public Health, Nagoya City University Graduate School of Medical Sciences, Nagoya, Japan; 11 Division of Cancer Prevention and Epidemiology, Chiba Cancer Center, Chiba, Japan; 12 Department of Public Health, Shiga University of Medical Science, Shiga, Japan; 13 Department of Food Science and Human Nutrition, Faculty of Agriculture, Ryukoku University, Kyoto, Japan; 14 Department of Epidemiology for Community Health and Medicine, Kyoto Prefectural University of Medicine, Kyoto, Japan; 15 Department of Environmental Medicine and Infectious Disease, Kyushu University, Fukuoka, Japan; 16 Center for Advanced Medicine and Clinical Research, Nagoya University Hospital, Nagoya, Japan; 17 Laboratory for Genotyping Development, Center for Integrative Medical Sciences, RIKEN, Kanagawa, Japan

## Abstract

Coffee is one of the most widely consumed beverages worldwide, and its role in human health has received much attention. Although genome-wide association studies (GWASs) have investigated genetic variants associated with coffee consumption in European populations, no such study has yet been conducted in an Asian population. Here, we conducted a GWAS to identify common genetic variations that affected coffee consumption in a Japanese population of 11,261 participants recruited as a part of the Japan Multi-Institutional Collaborative Cohort (J-MICC) study. Coffee consumption was collected using a self-administered questionnaire, and converted from categories to cups/day. In the discovery stage (*n* = 6,312), we found 2 independent loci (12q24.12–13 and 5q33.3) that met suggestive significance (*P* < 1 × 10^−6^). In the replication stage (*n* = 4,949), the lead variant for the 12q24.12–13 locus (rs2074356) was significantly associated with habitual coffee consumption (*P* = 2.2 × 10^−6^), whereas the lead variant for the 5q33.3 locus (rs1957553) was not (*P* = 0.53). A meta-analysis of the discovery and replication populations, and the combined analysis using all subjects, revealed that rs2074356 achieved genome-wide significance (*P* = 2.2 × 10^−16^ for a meta-analysis). These findings indicate that the 12q24.12-13 locus is associated with coffee consumption among a Japanese population.

## Introduction

Coffee is one of the most widely consumed beverages worldwide^1^. Recent national data from Japan have revealed that the average per capita consumption of coffee is about 11 cups/week^2^. The role of coffee in human health has received much attention^3^. In prospective cohort studies and meta-analysis studies, coffee consumption has been inversely associated with risk of stroke, cardiovascular disease, and multiple chronic diseases, such as Parkinson disease, diabetes, and liver, urine, prostate and colorectal cancers^4–10^. For most populations, coffee is one of the most highly caffeinated beverages consumed. Given that there is considerable inter-individual variability in preference for caffeine, it has been suggested that habitual caffeine consumption is influenced by genetic factors, in addition to cultural, psychosocial, or environmental factors (smoking)^11^. Twin studies among populations of European ancestry reported heritability estimates for caffeine use which ranged from 36% to 58%^12^. Five genome-wide association studies (GWAS) have been carried out on coffee or caffeine consumption^13–17^. The early GWASs discovered associations between coffee or caffeine consumption and several genes, namely *CYP1A1*-*CYP1A2*^13–15^, *AHR*^13,14^, *NRCAM* and *ULK3*^15^. A very large GWAS among nearly 130 thousand people confirmed the association with *CYP1A1*-*CYP1A2* and *AHR*, and also identified six novel loci, namely *ABCG2*, *POR*, *BDNF*, *SLC6A4*, *MLXIPL*, and *GCKR*^16^. Most recently, a GWAS revealed a significant association between *PDSS2* and habitual coffee consumption^17^. All these studies investigated subjects of European and/or African American ancestry, however, and no study has been conducted in an Asian population.

Here, we conducted a genome-wide association study to identify common genetic variations that affect coffee consumption in a Japanese population.

## Results

We analyzed the effects of common variants on coffee consumption from two study populations in the J-MICC study, namely 6,312 individuals for the discovery stage and 4,949 individuals for the replication stage. We also analyzed the 11,261 individuals (total of the two study populations) for the replication of SNPs previously reported in western populations. Baseline characteristics of these two groups of participants are shown in Table 1, and baseline characteristics of participants according to site are shown in Supplementary Table 1. Mean age in the discovery and replication populations was 53.0 ± 9.9 and 55.1 ± 8.8 years old, and percentage of female participants was 55% and 53%, respectively. Coffee consumption was 1.6 ± 1.5 cups/day for the discovery and 1.7 ± 1.5 cups/day for the replication populations.

**Table 1:** Baseline characteristics of the study subjects.

	Discovery stage	Replication stage	All subjects (Discovery and Replication stage)
Number	6,312	4,949	11,261
Female (%)	55.0	52.6	53.9
Age ± SD (year)	53.0 ± 9.9	55.1 ± 8.8	54.0 ± 9.5
Coffee consumption (mean ± SD) (cups/day)	1.6 ± 1.5	1.7 ± 1.6	1.7 ± 1.5
Current alcohol drinkers (%)	56.1	54.8	55.6
Current alcohol consumption (mean ± SD) (g/day)*	23.9 ± 28.5	27.0 ± 28.3	25.3 ± 28.5
BMI (kg/m2) (mean ± SD)	22.7 ± 3.2	23.4 ± 3.4	23.0 ± 3.3
Smoking status			
Current smokers (%)	17.4	20.5	18.8
Former smokers (%)	24.0	22.4	23.3
Never smokers (%)	58.5	57.1	57.9

*Among current alcohol consumers.

### Genome-wide association study in a Japanese population

#### Discovery stage

**Figure 1:**
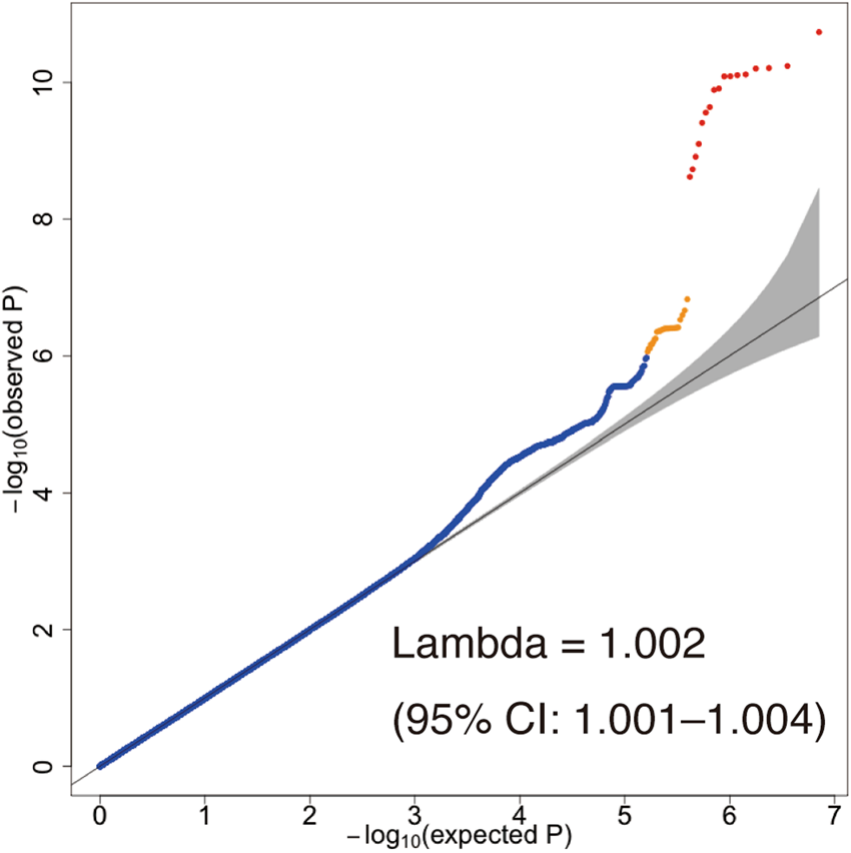
Quantile-quantile plot of genome-wide association tests using discovery samples (*N* = 6,312). The *x*-axis indicates the expected −log_10_ *P*-values under the null hypothesis. The *y*-axis shows the observed −log_10_ *P*-values calculated by a mixed linear model association method. The black line represents *y* = *x*, which corresponds to the null hypothesis. The gray shaded area shows 95% confidence intervals of the null hypothesis. The inflation factor (lambda) is the median of the observed test statistics divided by the median of the expected test statistics. Variants with *P*-values indicating less than suggestive significance (*P* < 1 × 10^−6^) and genome-wide significance (*P* < 5 × 10^−8^) are shown in orange and red, respectively.

**Figure 2:**
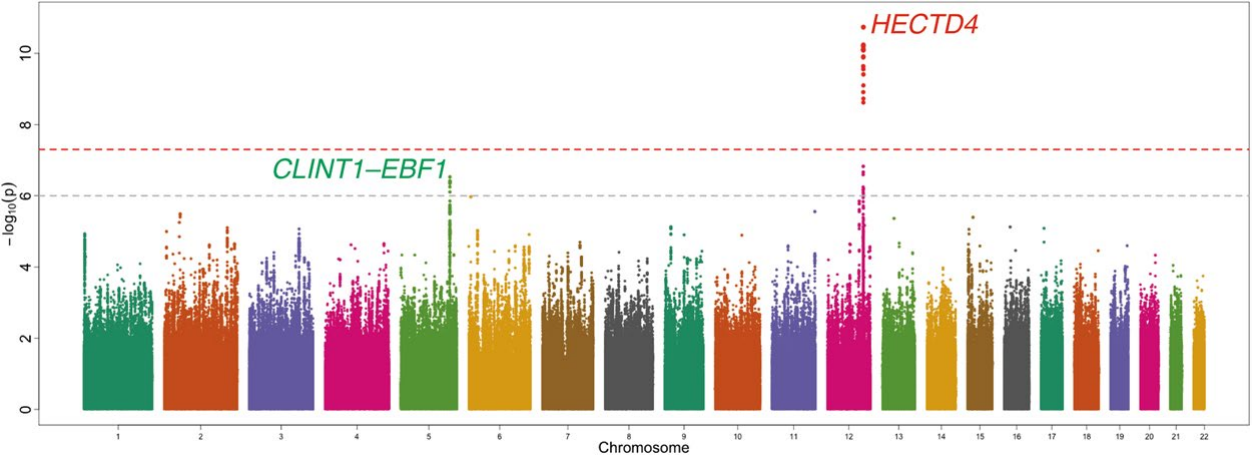
Genome-wide association signals from the discovery samples (*N* = 6,312). The *x*-axis represents chromosomal positions and the *y*-axis represents −log_10_ *P*-values calculated by a mixed linear model association analysis. The grey and red dotted horizontal lines indicate the suggestive (*P* = 1 × 10^−6^) and genome-wide (*P* = 5 × 10^−8^) significance levels, respectively. Variants with *P*-values indicating less than genome-wide significance (*P* < 5 × 10^−8^) are shown in red.

We performed a genome-wide scan for habitual coffee consumption-associated genetic variants based on discovery samples (*N* = 6,312) with adjustment for age and sex. The quantile-quantile plot of the observed *P* values is shown in Fig. 1. The inflation factor of the genome-wide scan was 1.002 (95% confidence interval: 1.001–1.004), indicating that the population structure was well-adjusted. Figure 2 shows scatter plots of *P* values derived from genome-wide scan results for coffee consumption, which found that two independent loci (12q24.12–13 and 5q33.3) met suggestive significance (*P* < 1 × 10^−6^) (Table 2). Genome-wide analyses adjusted for age, sex and smoking status and adjusted for age, sex, smoking status and BMI did not find any other loci achieving suggestive significance (Supplementary Tables 2 and 3). The association between 12q24.12–13 locus and habitual coffee consumption was not attenuated by modifying adjustment variables (Supplementary Tables 2 and 3).

**Table 2:** SNPs associated with habitual coffee consumption.

SNP	Chr^b^	Gene(s)	Position^c^	EA^d^	NEA^e^	Rsq^f^	Population	AF^g^	Beta^h^	SE (Beta)^i^	Variance explained (%)	*P*
rs1957553	5	*CLINT1–EBF1* (intergenic)	157,506,734	A	G	0.996	Discovery	0.272	0.1489	0.0290	0.42	3.0 × 10^−7^
							Replication	0.276	−0.0216	0.0348	0.01	5.3 × 10^−1^
							Meta-analysis	0.274	0.0789	0.0223	0.11	4.0 × 10^−4^
rs2074356^a^	12	*HECTD4* (intron)	112,645,401	A	G	0.996	**Discovery**	**0.252**	**0.2011**	**0.0299**	**0.73**	**1.8 × 10** ^**−11**^
							Replication	0.224	0.1777	0.0376	0.44	2.2 × 10^−6^
							**Meta-analysis**	**0.240**	**0.1920**	**0.0234**	**0.59**	**2.2 × 10** ^**−16**^

^a^Directly genotyped; ^b^Chromosome; ^c^Chromosomal position (GRCh37/hg19); ^d^Effect allele; ^e^Non-effect allele; ^f^Imputation quality in terms of R-square calculated by the Minimac3 software version 1.0.11; ^g^Allele frequency of effect allele; ^h^Effect size; ^i^Standard error of effect size Results listed in bold are the associations whose P-values are less than of genome-wide significance (P < 5 × 10^−8^).

**Figure 3:**
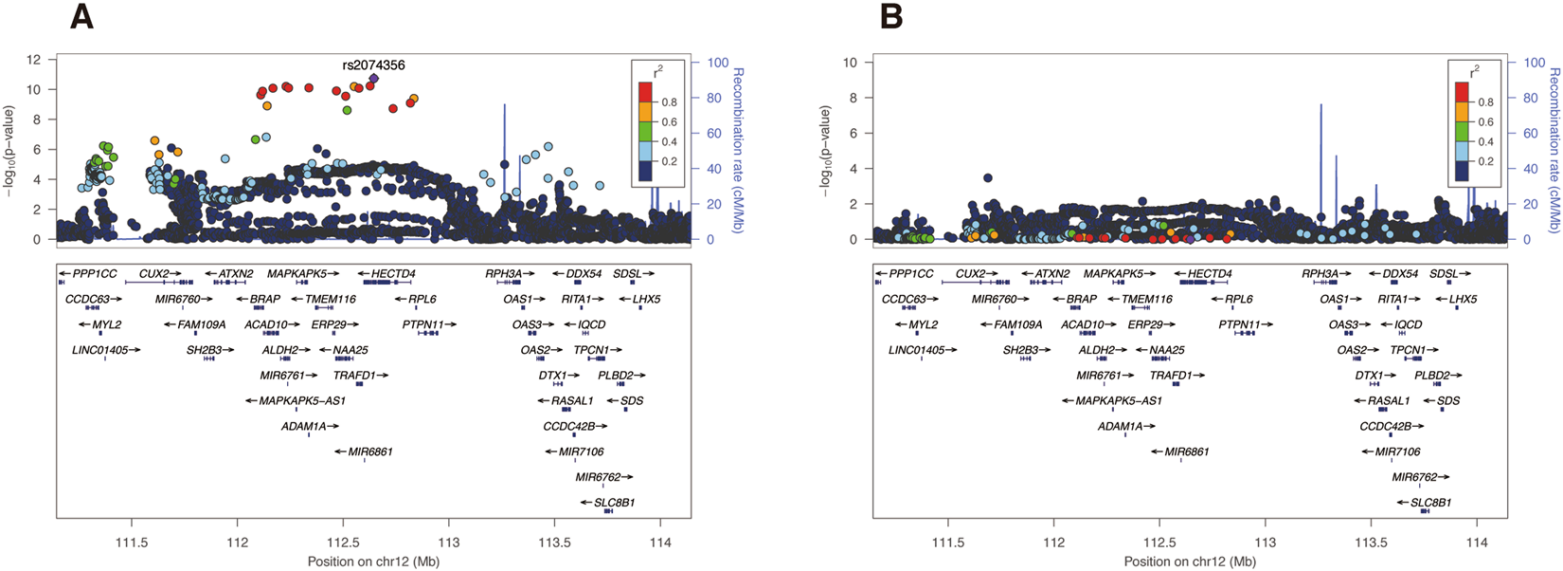
Association signals around the *HECTD4* gene using discovery samples (*N* = 6,312). The *x*-axis represents chromosomal positions near the *HECTD4* gene, and the *y*-axis represents −log_10_ *P*-values. The top signal in this locus (rs2074356) is shown in purple. Dot color for a variant represents the degree of linkage disequilibrium (*R*^2^) estimates between each variant and rs2074356. (**A**) Signals from a genome-wide association scan adjusted for age and sex. (**B**) Signals from conditional analysis adjusted for age, sex and rs2074356 dosage.

Regarding the 12q24.12–13 locus, the strongest significance was observed at rs2074356, which is located at an intron of the *HECTD4* gene (Fig. 3A). The rs2074356 A allele was significantly associated with high consumption of coffee (*P* = 1.8 × 10^−11^), and its effect size was estimated as 0.20 (standard error = 0.03) cups/day per allele. A conditional analysis showed that the association between 12q24 variants and habitual coffee consumption did not achieve suggestive significance when conditioned on the rs2074356 genotype (Fig. 3B and Supplementary Table 4). The frequency of the rs2074356 A allele was 25.2% in our discovery population. The rs2074356 A allele is East Asian-specific and monomorphic in Europeans, Africans, Americans, and South Asians according to the 1000 Genomes reference panel^18,19^.

The lead variant at 5q33.3 was rs1957553, which is located at an intergenic region between *CLINT1* and *EBF1*. The rs1957553 A allele was associated with a 0.15 (SE = 0.03) cups/day per allele increase in habitual coffee consumption (*P* = 3.0 × 10^−7^). The frequency of the rs1957553 A allele was 27.2% in our discovery population versus 61% in a European population of the 1000 Genomes reference panel^18,19^.

#### Replication stage and meta-analysis

In the analysis of replication samples (*N* = 4,949), rs2074356 was strongly associated with habitual coffee consumption (*P* = 2.2 × 10^−6^), but no significant association was seen for rs1957553 (*P* = 0.53). A meta-analysis of the discovery and replication populations revealed that rs2074356 achieved genome-wide significance (*P* < 5 × 10^−8^), but rs1957553 did not (Table 2). The phenotypic variance explained by rs2074346 was estimated at 0.59% from the meta-analysis.

In the meta-analysis, 24 variants in the 12q24.12–13 locus met genome-wide significance (Table 3). Of these 24 variants, 6 were located at intergenic regions; 15 were at intron regions; 1 was in the 3′ UTR; 1 was a synonymous variant; and 1 was a missense variant. The 1 missense variant was rs671, which is located in the *ALDH2* gene and has been associated with alcohol drinking^20^. We investigated the expression quantitative trait loci (eQTL) relationship between the 24 significant variants and surrounding genes (Table 3). From the GTEx database^21^, no eQTL hit was found, possibly because the 12q24.12–13 variants are monomorphic in European populations. Accordingly, we looked up the Human Genetic Variation Database^22^, which is based on Japanese data, and found 5 genes (*TRAFD1*, *ALDH2*, *HECTD4*, *MAPKAPK5*, and *RPH3A*) whose expression levels were nominally significantly associated with the 12q24.12–13 variants (*P* < 0.05; Table 3).

**Table 3:** Functional annotations for SNPs associated with habitual coffee consumption in 12q24 locus.

SNP	Chr^b^	Position^c^	Gene(s)	EA^d^	NEA^e^	Rsq^f^	AF^g^	Beta^h^	SE (Beta)^i^	Variance explained (%)	*P*	eQTL (*P* < 0.05)
rs12227162	12	111,367,244	*MYL2–CUX2* (intergenic)	T	C	0.974	0.201	0.1756	0.0252	0.44	3.2 × 10^−12^	no hit
rs149607519	12	111,389,437	*MYL2–CUX2* (intergenic)	G	C	0.993	0.206	0.1682	0.0247	0.41	1.0 × 10^−11^	no hit
rs148177611	12	111,390,454	*MYL2–CUX2* (intergenic)	T	TAGAA	0.987	0.208	0.1672	0.0247	0.41	1.3 × 10^−11^	no hit
rs3809297	12	111,609,727	*CUX2* (intron)	T	G	0.872	0.247	0.1670	0.0248	0.46	1.7 × 10^−11^	no hit
rs11065992	12	112,085,496	*BRAP* (intron)	C	T	0.804	0.461	0.1259	0.0225	0.35	2.3 × 10^−8^	no hit
rs3782886^a^	12	112,110,489	*BRAP* (synonymous)	C	T	0.996	0.277	0.1808	0.0224	0.58	6.7 × 10^−16^	*TRAFD1;ALDH2*;*HECTD4*;*MAPKAPK5*
rs11066001	12	112,119,171	*BRAP* (intron)	C	T	0.984	0.273	0.1835	0.0226	0.59	4.4 × 10^−16^	no hit
rs60125993	12	112,136,208	*ACAD10* (intron)	C	CT	0.906	0.502	0.1417	0.0209	0.44	1.2 × 10^−11^	no hit
rs11066008	12	112,140,669	*ACAD10* (intron)	G	A	0.828	0.342	0.1646	0.0232	0.54	1.5 × 10^−12^	no hit
rs11066015^a^	12	112,168,009	*ACAD10* (intron)	A	G	0.999	0.264	0.1829	0.0227	0.57	8.9 × 10^−16^	no hit
rs4646776	12	112,230,019	*ALDH2* (intron)	C	G	0.997	0.264	0.1843	0.0227	0.58	4.4 × 10^−16^	no hit
rs671^a^	12	112,241,766	*ALDH2* (missense)SIFT: deleterious PolyPhen: possibly damaging	A	G	0.999	0.264	0.1840	0.0227	0.58	4.4 × 10^−16^	*TRAFD1*; *MAPKAPK5*; *HECTD4*
rs78069066	12	112,337,924	*MAPKAPK5* (3′ UTR)	A	G	0.964	0.268	0.1874	0.0230	0.61	4.4 × 10^−16^	*TRAFD1*; *HECTD4*; *MAPKAPK5*; *ALDH2*
rs11066132	12	112,468,206	*NAA25* (intron)	T	C	0.929	0.260	0.1884	0.0236	0.60	1.3 × 10^−15^	no hit
rs116873087	12	112,511,913	*NAA25* (intron)	C	G	0.929	0.263	0.1876	0.0235	0.60	1.3 × 10^−15^	no hit
rs11066150	12	112,518,803	*NAA25* (intron)	A	G	0.802	0.454	0.1421	0.0225	0.44	2.7 × 10^−10^	no hit
rs147992802	12	112,552,274	*NAA25-TRAFD1* (intergenic)	T	C	0.803	0.337	0.1762	0.0236	0.61	8.1 × 10^−14^	no hit
rs12231737	12	112,574,616	*TRAFD1* (intron)	T	C	0.936	0.269	0.1894	0.0233	0.62	4.4 × 10^−16^	no hit
rs144504271	12	112,627,350	*HECTD4* (intron)	A	G	0.955	0.265	0.1895	0.0231	0.62	2.2 × 10^−16^	no hit
rs2074356^a^	12	112,645,401	*HECTD4* (intron)	A	G	0.996	0.240	0.1920	0.0234	0.59	2.2 × 10^−16^	*TRAFD1;MAPKAPK5*
rs77768175	12	112,736,118	*HECTD4* (intron)	G	A	0.818	0.244	0.1987	0.0256	0.64	8.9 × 10^−15^	no hit
rs11066280^a^	12	112,817,783	*HECTD4* (intron)	A	T	0.997	0.279	0.1742	0.0223	0.54	5.3 × 10^−15^	*RPH3A*; *TRAFD1*;*MAPKAPK5*
rs11537471	12	112,834,586	*HECTD4-RPL6* (intergenic)	G	A	0.843	0.340	0.1702	0.0229	0.57	1.1 × 10^−13^	no hit
rs139144808	12	113,470,025	*OAS2–DTX1* (intergenic)	TA	T	0.873	0.221	0.1433	0.0256	0.31	2.2 × 10^−8^	no hit

^a^Directly genotyped; ^b^Chromosome; ^c^Chromosomal position (GRCh37/hg19); ^d^Effect allele; ^e^Non-effect allele; ^f^Imputation quality in terms of R-square calculated by the Minimac3 software version 1.0.11; ^g^Allele frequency of effect allele; ^h^Effect size; ^i^Standard error of effect size.

#### Combined analysis of discovery and replication subjects

The above-mentioned analyses employed a discovery-replication scheme, which is useful for avoiding false positive associations caused by confounding factors, such as population stratification. We also investigated if additional loci associated with habitual coffee consumption might be suggested from our Japanese data using genome-wide association tests including both discovery and replication subjects (*N* = 11,261), with three sets of adjustment variables: (i) age and sex, (ii) age, sex, and smoking status, and (iii) age, sex, smoking status, and BMI. The results showed that only the 12q24.12–13 locus achieved genome-wide significance (Supplementary Table 5 and Supplementary Figure 1). An additional three loci had suggestive significance (Supplementary Table 5 and Supplementary Figure 1). Of these three loci, the *AGR3–AHR* locus had been associated with habitual caffeine or coffee consumption in previous GWASs^13,14,16^. The other two loci, *CT49–DNAH5* and *MAB21L3–ATP1A1*, were not reported in previous GWASs, and were therefore considered to be novel candidate loci potentially associated with habitual coffee consumption.

#### Confounding factor adjustment

Rs 671, one of the significant 12q24 variants, is well known to be a functional polymorphism in the *ALDH2* gene that affects the activity of ALDH2 in East Asian populations^20^. Reduced activity of ALDH2 is associated with increased concentrations of the toxin acetaldehyde and manifestation of the alcohol flush reaction, which protects individuals with the *ALDH2* 504Lys variant(s) from heavy drinking^23^. We estimated the association between rs2074356 and coffee consumption adjusted for alcohol consumption in the 11,261 individuals (total of the two study populations). In the additive model, the rs2074356 A allele was significantly associated with high coffee consumption after adjustment for age, sex and alcohol consumption (*β* = 0.176, *P* < 0.001). We also estimated the association between rs2074356 and coffee consumption in the 11,261 individuals when stratified into alcohol drinkers and non-drinkers. In alcohol drinkers, the rs2074356 A allele was significantly associated with high coffee consumption after adjustment for age, sex and alcohol consumption (*β* = 0.357, p < 0.001). In non-alcohol drinkers, the rs2074356 A allele was significantly associated with high coffee consumption after adjustment for age and sex (*β* = 0.119, *P* < 0.001). Previous studies suggested the association of rs2074356 in *HECTD4* with body mass index (BMI)^24^. A recent GWAS proposed that the minor allele of the *HECTD4* variant rs2074356 is associated with a recused Thoracic-to-Hip ratio^25^, which relates to BMI level. We estimated the association between rs2074356 and coffee consumption adjusted for BMI levels in the 11,261 individuals. The rs2074356 A allele was significantly associated with high coffee consumption after adjustment for age, sex and BMI levels (*P < *0.001). A recent study found that the *ALDH2* 504Lys variant(s) is associated with smoking initiation^26^. Smoking is a factor associated with caffeine consumption^11^. We estimated the association between rs2074356 and coffee consumption adjusted for smoking status in the 11,261 individuals. The rs2074356 A allele was significantly associated with high coffee consumption after adjustment for age, sex and smoking status (*P* < 0.001). In a regression analysis adjusted for age, sex, alcohol, BMI levels and smoking status, the rs2074356 A allele was significantly associated with high consumption of coffee (*β* = 0.147, *P* < 0.001 for overall, *β* = 0.32, *P* < 0.001 for alcohol drinkers, *β* = 0.092, *P* = 0.002 for non-alcohol drinkers).

#### Replication of previously reported SNPs in the Japanese population

The 5 GWASs on coffee or caffeine consumption described to date^13–17^ have reported 18 SNPs (Supplementary Table 6). All 5 previous GWASs were conducted in individuals of European and/or African American ancestry. Variants on 7p21 (rs4410790 and rs6968554) and 15q24 (rs2470893 and rs2472297) were well-replicated, mainly in the European populations^13,27,28^. In the J-MICC population of Japanese, in contrast, rs2470893 and rs2472297 were monoallelic. Variants on 6q21 showed very low minor allele frequencies (MAFs) (<0.002). Table 4 shows the associations between the remaining 11 SNPs and habitual coffee consumption with adjustment for age and sex. Six variants (rs1260326, rs4410790, rs6968554, rs6968865, rs17685 and rs6265) were nominally significant (*P* < 0.05), while three variants on 7p21 (rs4410790, rs6968554 and rs6968865) were significant after multiple correction (*P* < 0.05/11). We estimate how much phenotypic variance in coffee consumption could be explained by the SNPs identified in Table 4. The explained variance ranged between 0.05% and 0.19%. The effect directions of all nominally significant variants were consistent with previous GWASs^13–17^ (Table 4).

**Table 4:** Replication analysis using the J-MICC samples for previously-reported SNPs.

SNP	Chr^b^	Position^c^	Gene(s)	EA^d^	NEA^e^	Rsq^f^	AF^g^	Beta^h^	SE (Beta)^i^	Variance explained (%)	*P*	Effect direction in European GWAS^j^
**rs1260326** ^**a**^	**2**	**27,730,940**	***GCKR*** **(missense) SIFT: tolerated PolyPhen: benign**	**C**	**T**	**0.996**	**0.442**	**0.0476**	**0.0207**	**0.05**	**0.02**	**+**
rs1481012	4	89,039,082	*ABCG2* (intron)	A	G	0.975	0.704	0.0339	0.0226	0.02	0.13	+
**rs4410790** ^**a**^	**7**	**17,284,577**	***AGR3–AHR*** **(intergenic)**	**C**	**T**	**0.996**	**0.375**	**0.0959**	**0.0207**	**0.19**	**3.7 × 10** ^**−6**^	**+**
**rs6968554** ^**a**^	**7**	**17,287,106**	***AGR3–AHR*** **(intergenic)**	**G**	**A**	**0.999**	**0.341**	**0.0908**	**0.0212**	**0.16**	**1.9 × 10** ^**−5**^	**+**
**rs6968865** ^**a**^	**7**	**17,287,269**	***AGR3–AHR*** **(intergenic)**	**T**	**A**	**1.000**	**0.340**	**0.0908**	**0.0212**	**0.16**	**1.9 × 10** ^**−5**^	**+**
rs7800944	7	73,035,857	*MLXIPL* (intron)	C	T	0.930	0.114	0.0430	0.0331	0.02	0.19	+
**rs17685** ^**a**^	**7**	**75,616,105**	***POR*** **(3′ UTR)**	**A**	**G**	**0.999**	**0.363**	**0.0554**	**0.0209**	**0.06**	**0.008**	**+**
rs382140^a^	7	107,782,200	*LAMB4–NRCAM* (intergenic)	A	G	0.997	0.254	0.0302	0.0230	0.02	0.19	+
**rs6265** ^**a**^	**11**	**27,679,916**	**BDNF (missense)SIFT: tolerated PolyPhen: probably damaging**	**C**	**T**	**1.000**	**0.592**	**0.0460**	**0.0206**	**0.05**	**0.03**	**+**
rs6495122^a^	15	75,125,645	*CPLX3–ULK3* (intergenic)	A	C	1.000	0.808	0.0068	0.0257	0.00	0.79	—
rs9902453	17	28,349,095	*EFCAB5* (intron)	A	G	0.983	0.338	0.0072	0.0218	0.00	0.74	—

^a^Directly genotyped; ^b^Chromosome; ^c^Chromosomal position (GRCh37/hg19); ^d^Effect allele; ^e^Non-effect allele; ^f^Imputation quality in terms of R-square calculated by the Minimac3 software version 1.0.11; ^g^Allele frequency of effect allele; ^h^Effect size; ^i^Standard error of effect size; ^j^Consistent direction is denoted as ‘+’ and inconsistent direction is denoted as ‘−’

Results listed in bold are associations whose P-value is less than genome-wide significance (P < 5 × 10^−8^).

## Discussion

In this study, we conducted the first GWAS on coffee intake in an Asian population. Participants were 6,312 individuals from a Japanese cohort study. Replication was attempted in another 4,949 individuals from the same cohort. A meta-analysis of the discovery and replication populations, we discovered that 24 novel SNPs on a 12q24 locus had genome-wide significance with habitual coffee consumption. The 24 SNPs associated with coffee intake were located at 13 genes, namely the *ALDH2*, *ACAD10*, *BRAP*, *ADAM1A*, *NAA25*, *TRAFD1*, *RPL6*, *MYL2, CUX2, OAS2, DTX1, MAPKAPK5* and *HECTD4* regions on the 12q24.12-13. Because these genes showed strong linkage disequilibrium, our results suggest that the 12q24.12-13 locus is responsible for variations in coffee consumption. We also confirmed an association between coffee intake and 6 SNPs previously reported in western populations.

Associations of the discovered genes with coffee consumption are intriguing but have not been studied well. One of these SNPs associated with coffee, rs 671, is a missense mutation and a functional Glu504Lys polymorphism in the *ALDH2* gene, namely a substitution of the Glu at codon position 504 with Lys, which affects the activity of ALDH2^20^. The reduced activity of ALDH2, shown with the *ALDH2* 504Lys variant(s), contributes to increasing blood concentrations of the toxic acetaldehyde and exhibition of the alcohol flush reaction that protects individuals with the *ALDH2* 504Lys variant(s) from heavy drinking. One study found that coffee consumption was higher with the *ALDH2 504Lys* allele in Japanese men^29^, which was consistent with our result. The *ALDH2* 504Lys variant(s) is associated with smoking initiation^26^, and smoking is associated with caffeine consumption^11^. Rs2074356 is located at an intron of the *HECTD4* gene. *HECTD4* may encode E3 ubiquitin protein ligase, which is a member of the ubiquitin ligase family. E3 ligases is involved in the final step in the ubiquitination cascade, catalyzing transfer of ubiquitin from an E2 enzyme to form a covalent bond with a substrate lysine^30^. rs2074356 in *HECTD4* has been associated with drinking behavior in Han Chinese^31^. We confirmed that the association of rs2074356 in *HECTD4* with coffee was not attenuated by adjustment for alcohol consumption. We also confirmed that the association was not attenuated by adjustment for smoking status. All these discovered genes showed strong linkage disequilibrium. The results suggest that the association of the 12q24.12-13 locus with coffee consumption is not confounded by alcohol drinking or smoking status. Previous studies have reported associations of this 12q24 region with metabolic syndrome, thoracic-to-hip ratio^25^, kidney function^32^, and BMI levels^33^. Coffee consumption is also reported to be inversely associated with BMI levels^34^. Our results indicate that the 12q24 region is independently associated with coffee consumption after adjustment for BMI level. Although our results do not allow us to conclude which is SNP is the most closely associated with coffee consumption, evidence from our GWAS suggests that the 12q24.12-13 locus is strongly associated with habitual coffee consumption. Because the SNPs of genes in this study exist only in East Asians, their association with coffee consumption in western populations must be rare. Identified SNPs are associated with the expression level of *ALDH2, HECTD4, TRAFD1, MAPKAPK5* and *RPH3A*. TRAFD1 (TRAF-Type Zinc Finger Domain Containing 1), encoded by *TRAFD1*, is a negative feedback regulator that controls excessive immune responses^35^. MAPKAPK5 (Mitogen-Activated Protein Kinase-Activated Protein Kinase 5), encoded by *MAPKAPK5*, is a tumor suppressor and member of the serine/threonine kinase family^36^. In response to cellular stress and proinflammatory cytokines, this kinase is activated through its phosphorylation by MAP kinases, including MAPK1/ERK, MAPK14/p38-alpha, and MAPK11/p38-beta^36^. RPH3A encoded by *RPH3A* is thought to be an effector for RAB3A, which is a small GTP-binding protein that acts in neurotransmitter exocytosis^37^. Genome-wide association tests using both discovery and replication subjects identified three loci with suggestive significance, among which the *AGR3–AHR* locus was shown to be associated with habitual caffeine or coffee consumption in previous GWASs^13,14,16^. The aryl hydrocarbon receptor (AHR), encoded by *AHR*, is a ligand-activated transcription factor that induces genes encoding CYP1A1 and CYP1A2^38^, of which CYP1A2 is involved in the metabolism of widely used drugs and is a caffeine-metabolized enzyme^39^.

In Japan, the most popular types of coffee are instant coffee, brewed coffee and canned coffee^2^. Brewed coffee is made by brewing hot water with ground coffee beans. Brewing is most commonly done by drip or filter, and less commonly under pressure with an espresso machine. Several limitations of this study warrant mention. First, we did not evaluate details of coffee intake, such as cup size, use of caffeinated or decaffeinated coffee, or method of preparation (filtered or boiled). However, decaffeinated coffee and boiled coffee are very uncommon in Japan, and it was considered that assessment of the use and methods of coffee consumption and evaluation of their effects among Japanese would be uninformative. Second, because only a small number of participants (<5% of total participants) were cancer patients, they tended to underreport past coffee consumption as a result of decreased dietary intake. We minimized this limitation by asking these patients about their lifestyle when they were healthy or before their current symptoms developed. However, because more than 95% of the study participants were from a healthy general population, we consider that this study has external validity for the general Japanese population. Lastly, most of the functional effects of these coffee consumption-associated SNPs, including rs2074356, remain unclear. The functional relevance of the identified SNPs to coffee consumption remains to be determined. Therefore, our findings warrant further functional study to support the observed association between variants in the 12q24.12-13 locus and coffee consumption.

Coffee consumption is well known to be associated with a reduced risk of stroke, cardiovascular disease, Parkinson disease, diabetes, as well as liver, urine, prostate and colorectal cancers^4–10^. However, the genetic factors associated with coffee have never been considered in the association of health benefits with coffee intake. Adjustment for the genetic factors found in this study should aid in establishing the association between coffee consumption and health benefits. Our study indicates the need for further research to evaluate the effect of genetic factors and coffee consumption on the relationship between coffee consumption and health outcomes.

In conclusion, we have discovered that the 12q24.12-13 locus is associated with coffee consumption among a Japanese population. This is the first report to identify a SNP for coffee consumption in an Asian population. Further studies are needed to investigate the biological mechanism that links the 12q24.12-13 locus and coffee consumption.

## Methods

### Study population

The GWAS was conducted in participants aged 35-69 years as a cross-sectional study within the Japan Multi-Institutional Collaborative Cohort (J-MICC) study. The 14,539 subjects of the J-MICC study were recruited from 12 different areas throughout Japan (Chiba, Okazaki, Shizuoka-Daiko, Takashima, Kyoto, Sakuragaoka, Aichi, Saga, Kagoshima, Tokushima, Fukuoka and Kyushu-KOPS) between 2004 and 2013. The 2,830 participants from two areas (Fukuoka and Kyushu-KOPS) were excluded from this study because the questionnaire on habitual coffee consumption in these areas was inconsistent with that used in the other 10 areas. Subjects who did not answer the questionnaire on habitual coffee consumption were also excluded. After quality control filtering (described below), a total of 11,261 participants were used in this study. For the discovery stage, we used the samples from the 6,312 participants from the 6 areas of Chiba, Okazaki, Shizuoka-Daiko, Takashima, Kyoto and Sakuragaoka. For the replication stage, we used the 4,949 participants from the 4 areas of Aichi, Saga, Kagoshima and Tokushima. The J-MICC study is a large cohort study launched in 2005 to confirm gene environment interactions in lifestyle-related disease. Details of the J-MICC Study have been reported elsewhere^40^. Briefly, participants completed a questionnaire about lifestyle and medical information, and donated a blood sample at the time of the baseline survey. The J-MICC study participants included community citizens, first-visit patients to a cancer hospital and health check examinees. All participants in this study gave written informed consent, and the study protocol was approved by the Ethics Committees of Aichi Cancer Center, Nagoya University Graduate School of Medicine, and the other institutions participating in the J-MICC study. The present study was conducted according to the principles expressed in the World Medical Association Declaration of Helsinki.

### Phenotype

The questionnaire for the J-MICC studies included questions on medical history, height, weight, family history (parents and siblings), smoking and drinking habits, dietary habits, sleeping habits, physical exercise and reproductive history. All exposures were collected using a scientifically validated self-administered questionnaire^41,42^. The questionnaire was checked by trained staff to ensure completeness and consistency. Information on coffee was obtained in terms of frequency and intake from seven categories (never, <2 cups/week, 3–4 cups/week, 5–6 cups/week, 1–2 cup/day, 3–4 cups/day, ≥5 cups/day), for each of two types of coffee (drip, filter or instant) and (canned, plastic bottled, or carton). Canned coffee is a ready-to-drink canned coffee beverage which is very popular in Japan. The coffee categories were converted to cups/day by taking the median value of each category. Total coffee consumption was estimated as the sum amount of the two coffee types.

### Genotyping and quality control filtering

Buffy coat fractions and DNA were prepared from blood samples and stored at −80 °C at the central J-MICC Study office. DNA was extracted from all buffy coat fractions using a BioRobot M48 Workstation (Qiagen Group, Tokyo, Japan) at the central study office. For the samples from two areas (Fukuoka and Kyushu-KOPS), DNA was extracted locally from samples of whole blood using an automatic nucleic acid isolation system (NA-3000, Kurabo, Co., Ltd, Osaka, Japan). The 14,539 study participants from the 12 areas of the J-MICC study, which includes the discovery and replication subjects, were genotyped at RIKEN Center for Integrative Medicine Sciences using a HumanOmniExpressExome-8 v1.2 BeadChip array (Illumina Inc., San Diego, CA, USA). Twenty-six samples with inconsistent sex information between the questionnaire and an estimate from genotyping were excluded. The identity-by-descent method implemented in the PLINK 1.9 software^43,44^ identified 388 close relationship pairs (pi-hat > 0.1875) and one sample of each pair was excluded. Principal component analysis (PCA)^45,46^ with a 1000 Genomes reference panel (phase 3)^18,19^ detected 34 subjects whose estimated ancestries were outside the Japanese population^47^. These 34 samples were excluded. The remaining 14,091 samples all met a sample-wise genotype call rate criterion (≥0.99). SNPs with a genotype call rate <0.98 and/or a Hardy-Weinberg equilibrium exact test *P-*value < 1 × 10^−6^ were removed, resulting in 873,254 autosomal variants. Of these, 298,644 variants with a low minor allele frequency (MAF) < 0.01 were excluded. This quality control filtering resulted in 14,091 individuals and 574,423 SNPs. Of the 14,091 samples, 6,312 were from the 6 areas for discovery analysis and 4,949 were from the 4 areas for replication analysis. The replication samples were subjected to genome-wide genotyping, followed by genome-wide imputation. However, only candidate novel loci identified during the discovery analysis were examined in the replication analysis to avoid the risk of identifying false-positive associations. A total of 11,261 samples from the 10 areas were also analyzed for replication of the previously reported SNPs in western populations. In addition to the discovery and replication design, we additionally conducted combined analysis of all the discovery and replication subjects (*N* = 11,261) to determine if our Japanese data might indicate additional loci associated with habitual coffee consumption.

### Genotype imputation

Genotype imputation was performed using SHAPIT^48^ and Minimac3^49^ software based on the 1000 Genomes reference panel (phase 3)^18^. After genotype imputation, strict quality control filters were applied; namely, variants with an R^2^ < 0.8^50^ and a MAF < 0.01 were excluded, resulting in 7,094,228 variants.

### Association tests between genetic variants and habitual coffee consumption

The association between genetic variants and quantitative habitual coffee consumption was tested using the mixed linear model association (MLMA) method^51^ with adjustment for age and sex. The mixed linear model uses adjustment covariates as fixed-effect variables and a genetic relationship matrix (GRM) as a variance-covariance matrix for random effects. To calculate the GRM, the genotyped SNPs were excluded using the quality-control criteria proposed in a previous study (genotype call rate ≥ 0.95, Hardy-Weinberg exact test *P*-value ≥ 0.05, and minor allele frequency ≥ 0.01)^52^, and the remaining 482,567 SNPs on autosomal chromosomes were used. Calculation of the GRM and genome-wide association tests were performed with the GCTA software^53^ version 1.24.2. An advantage of the MLMA method over a linear regression method adjusted for principal components is its prevention of false positive associations due to population or relatedness structure^54^. Because the Japanese population structure is not perfectly homogenous^47^ and previous Japanese GWASs adjusted for principal components, they reported genomic inflation factor values which were slightly higher than expected (>1.0)^55–57^. Accordingly, we chose the MLMA method to avoid the detection of false positive associations.

In the discovery stage, associations between all imputed variants and habitual coffee consumption was tested with adjustment for age and sex. This adjustment is consistent with 3 of 5 previous GWASs^13,15,17^. For variants achieving suggestive significance (*P* < 1 × 10^−6^) in the discovery stage, the associations with habitual coffee consumption were examined in the replication stage. We then combined the resulting summary statistics from the discovery and replication stages by using a fixed-effect model and inverse-variance weighting method for meta-analysis^58^. Variants achieving genome-wide significance (*P* < 5 × 10^−8^) in the meta-analysis were considered to be habitual coffee consumption-associated variants. In 2 of 5 previous GWASs^14,16^, smoking status was used as an adjustment variable. Accordingly, we additionally adjusted for smoking status in the discovery, replication and meta-analysis as a sensitivity analysis. Furthermore, we conducted discovery, replication and meta-analysis with adjustment for age, sex, smoking status and BMI because coffee consumption was reported to be inversely associated with BMI level^34^ and BMI can be a confounding factor of the genetic association with habitual coffee consumption.

For variants achieving genome-wide significance in the meta-analysis, we conducted conditional analysis based on the discovery population. In the conditional analysis, we tested the association between each variant and habitual coffee consumption by MLMA with adjustment for age, sex, and dosage of lead variant.

For replication analysis of previously reported SNPs, samples from the 10 areas were used for the association tests (*N* = 11,261).

### Functional annotations

We examined genomic locations of variants identified in this study based on the Ensembl^59^ and UCSC^60^ genome browsers. For missense variants, we looked up the Ensembl genome browser for bioinformatics prediction results from SIFT^61^ and PolyPhen^62^. *Cis*-eQTL pairs of variants and genes were obtained from the GTEx^21^ and Human Genetic Variation databases^22^.

### Confounding factor adjustment

Total alcohol consumption was estimated as the summed amount of pure alcohol consumption. The frequency of alcohol consumption was obtained in six categories (none, 1–3 times/month, 1–2 times/week, 3–4 times/week, 5–6 times/week, and everyday). Non-alcohol drinkers were defined as those who consumed alcohol none and 1–3 times/month. Alcohol drinkers were defined as those who consumed alcohol more than once/week. Smoking status was entered under the three categories of none, former, and current smoking. Multivariate linear regression analysis was used to test associations between SNP and coffee consumption with an additive model adjusted for age (continuous), sex, alcohol consumption (g/day) and BMI (continuous). Multivariate linear regression analysis was also used to test the association between SNP and coffee consumption with an additive model adjusted for age (continuous), sex, alcohol consumption (g/day) and BMI (continuous) according to alcohol drinking status (non-alcohol drinker and alcohol drinker). Values of *p* < 0.05 were considered statistically significant. The analyses were performed with Stata v. 14.1 (STATA Corporation, College Station, TX, USA).

### Data Availability Statement

The datasets generated during and/or analysed during the current study are not publicly available due to ethical restriction, but are available from the co-author on reasonable request.

## Electronic supplementary material

Supplementary Information
