# Supplementary material for: A genome-wide association study in the Japanese population identifies the 12q24 locus for habitual coffee consumption: The J-MICC Study
Source: Sci Rep. 2018 Jan 24;8:1493. doi: 10.1038/s41598-018-19914-w (PMC5784172; doi:10.1038/s41598-018-19914-w)
Supplement: Supplementary file 1 — Supplementary Information [file 41598_2018_19914_MOESM1_ESM.pdf]

A genome-wide association study in the Japanese population identifies the 12q24 locus for habitual coffee consumption: The J-MICC Study

Hiroko Nakagawa-Senda[1,2,10], Tsuyoshi Hachiya[3], Atsushi Shimizu[3], Satoyo Hosono[1], Isao Oze[4], Miki, Watanabe[1], Keitaro Matsuo[4,5], Hidemi Ito[4,5], Megumi Hara[6], Yuichiro Nishida[6], Kaori Endoh[7], Kiyonori Kuriki[7], Sakurako Katsuura-Kamano[8], Kokichi Arisawa[8], Yora Nindita[9], Rie Ibusuki[9], Sadao Suzuki[10], Akihiro Hosono[10], Haruo Mikami[11], Yohko Nakamura[11], Naoyuki Takashima[12], Yasuyuki Nakamura[13], Nagato Kuriyama[14], Etsuko Ozaki[14], Norihiro Furusyo[15], Hiroaki Ikezaki[15,] Masahiro Nakatochi[16], Tae Sasakabe[2], Sayo Kawai[2], Rieko Okada[2], Asahi Hishida[2], Mariko Naito[2], Kenji Wakai[2], Yukihide Momozawa[17], Michiaki Kubo[17], Hideo Tanaka[1]

[1]Division of Epidemiology and Prevention, Aichi Cancer Center Research Institute

[2]Department of Preventive Medicine, Nagoya University Graduate School of Medicine

[3]Division of Biomedical Information Analysis, Iwate Tohoku Medical Megabank Organization, Disaster Reconstruction Center, Iwate Medical University

[4]Division of Molecular and Clinical Epidemiology, Aichi Cancer Center Research Institute

[5]Department of Epidemiology, Nagoya University Graduate School of Medicine

[6]Department of Preventive Medicine, Faculty of Medicine, Saga University

[7]Laboratory of Public Health, Division of Nutritional Sciences, School of Food and Nutritional Sciences, University of Shizuoka

[8]Department of Preventive Medicine, Institute of Biomedical Sciences, Tokushima University Graduate School

[9]Department of International Island and Community Medicine, Kagoshima University Graduate School of Medical and Dental Sciences

[10]Department of Public Health, Nagoya City University Graduate School of Medical Sciences

[11]Division of Cancer Prevention and Epidemiology, Chiba Cancer Center

[12]Department of Public Health, Shiga University of Medical Science

[13]Department of Food Science and Human Nutrition, Faculty of Agriculture, Ryukoku University

[14]Department of Epidemiology for Community Health and Medicine, Kyoto Prefectural University of Medicine

[15] Department of Environmental Medicine and Infectious Disease, Kyushu University

[16]Center for Advanced Medicine and Clinical Research, Nagoya University Hospital

[17]Laboratory for Genotyping Development, Center for Integrative Medical Sciences, RIKEN

Supplement Table 1. Baseline characteristics of the study subjects according to site

Site	N	Age ± SD (year)	Female (%)	Coffee consumption (mean±SD) (cups/day)	Current alcohol drinkers (%)	Current alcohol consumption (mean±SD)	BMI (kg/m2) (mean±SD)	Current smoking rate (%)
Chiba	1,055	53.6 ± 9.9	66	1.5 ± 1.4	56.1	12.6 ± 22.8	22.5 ± 3.0	11.5
Okazaki	1,064	55.7 ± 9.1	45	1.6 ± 1.4	54.0	13.3 ± 22.4	22.4 ± 3.2	20.0
Shizuoka–Daiko	1,989	52.9 ± 9.7	56	1.6 ± 1.5	52.9	14.2 ± 29.8	23.3 ± 3.1	15.8
Takashima	543	56.5 ± 9.7	71	1.5 ± 1.4	55.9	13.1 ± 23.8	22.3 ± 3.1	15.7
Kyoto	1,088	49.8 ± 9.8	52	1.9 ± 1.6	46.6	10.4 ± 19.4	23.0 ± 3.3	20.0
Sakuragaoka	573	50.1 ± 9.4	38	1.5 ± 1.5	62.8	15.0 ± 23.7	22.4 ± 3.2	25.8
Sub-total	6,312	53.0 ± 9.9	55	1.6 ± 1.5	56.1	23.9 ± 28.5	22.7 ± 3.2	17.4
Aichi	1,150	55.1 ± 9.5	51	1.7 ± 1.5	56.3	13.4 ± 23.4	23.1 ± 3.2	21.7
Saga	1,897	56.8 ± 8.2	57	1.6 ± 1.6	51.8	18.1 ± 29.4	24.6 ± 3.6	20.8
Kagoshima	1,224	55.2 ± 8.3	58	1.7 ± 1.6	58.3	15.3 ± 23.4	23.9 ± 3.6	15.9
Tokushima	678	50.4 ± 8.9	33	2.2 ± 1.7	60	14.6 ± 23.7	23.1 ± 3.4	26.0
Sub-total	4,949	55.1 ± 8.8	53	1.7 ± 1.6	54.8	27.0 ± 28.3	23.4 ± 3.4	20.5

*Among current alcohol consumers

Supplementary Table 2. Results of genome-wide association study adjusted for age, sex and smoking status

SNP	Chr[b]	Gene	Position[c]	EA[d]	NEA[e]	Rsq[f]	Population	AF[g]	Beta[h]	SE(Beta)[i]	Variance explained (%)	*P*
rs2074356[a]	12	*HECTD4*	112,645,401	A	G	0.996	**Discovery**	**0.252**	**0.2084**	**0.0293**	**0.82**	**1.2×10^{-12}**
		(intron)					Replication	0.224	0.1681	0.0363	0.42	3.6×10^{-6}
							**Meta-analysis**	**0.240**	**0.1925**	**0.0228**	**0.62**	**3.1×10^{-17}**

[a] Directly genotyped; [b] Chromosome; [c] Chromosomal position (GRCh37/hg19); [d] Effect allele; [e] Non-effect allele; [f] Imputation quality in terms of R-square calculated by the Minimac3 software version 1.0.11; [g] Allele frequency of effect allele; [h] Effect size; [i] Standard error of effect size

Results listed in bold are associations whose *P*-values are less than of genome-wide significance ($P < 5\times10^{-8}$).

Supplementary Table 3. Results of genome-wide association study adjusted for age, sex , smoking status, and BMI

SNP	Chr[b]	Gene(s)	Position[c]	EA[d]	NEA[e]	Rsq[f]	Population	AF[g]	Beta[h]	SE(Beta)[i]	Variance explained (%)	*P*
rs1957553	5	*CLINT1–EBF1*	157,506,734	G	A	0.996	Discovery	0.2719	0.1394	0.0285	0.39	9.9×10^{-7}
		(intergenic)					Replication	0.2756	-0.0174	0.0340	0.01	6.1×10^{-1}
							Meta-analysis	0.2735	0.0746	0.0218	0.10	6.3×10^{-4}
rs2074356[a]	12	*HECTD4*	112,645,401	A	G	0.996	**Discovery**	**0.2520**	**0.2078**	**0.0293**	**0.82**	**1.4×10^{-12}**
		(intron)					Replication	0.2243	0.1674	0.0363	0.41	4.0×10^{-6}
							**Meta-analysis**	**0.2398**	**0.1918**	**0.0228**	**0.62**	**4.0×10^{-17}**

[a] Directly genotyped; [b] Chromosome; [c] Chromosomal position (GRCh37/hg19); [d] Effect allele; [e] Non-effect allele; [f] Imputation quality in terms of R-square calculated by the Minimac3 software version 1.0.11; [g] Allele frequency of effect allele; [h] Effect size; [i] Standard error of effect size

Results listed in bold are associations whose *P*-values were less than of genome-wide significance ($P < 5\times10^{-8}$).

Supplementary Table 4. Results of conditional analysis around rs2074356

SNP	Chr[b]	Position[c]	EA[d]	NEA[e]	AF[f]	Adjusted for age and sex			Adjusted for age, sex and rs2074356 dosage			LD[i] R^2
						Beta[g]	SE(Beta)[h]	*P*	Beta	SE(Beta)	*P*	
rs12227162	12	111,367,244	T	C	0.208	0.1616	0.0323	5.7×10^{-7}	0.0087	0.0322	0.787	0.522
rs149607519	12	111,389,437	G	C	0.213	0.1575	0.0317	6.8×10^{-7}	0.0081	0.0316	0.798	0.521
rs148177611	12	111,390,454	T	TAGAA	0.215	0.1574	0.0317	6.8×10^{-7}	0.0088	0.0315	0.781	0.515
rs3809297	12	111,609,727	T	G	0.256	0.1643	0.0319	2.5×10^{-7}	-0.0065	0.0317	0.837	0.657
rs3809284	12	111,688,139	C	T	0.906	0.2338	0.0473	7.9×10^{-7}	0.1691	0.0472	0.0003	0.037
rs11065992	12	112,085,496	C	T	0.473	0.1509	0.0291	2.2×10^{-7}	0.0209	0.0290	0.471	0.441
rs3782886[a]	12	112,110,489	C	T	0.288	0.1822	0.0287	2.3×10^{-10}	0.0076	0.0286	0.791	0.816
rs11066001	12	112,119,171	C	T	0.284	0.1867	0.0290	1.3×10^{-10}	0.0074	0.0289	0.798	0.842
rs60125993	12	112,136,208	C	CT	0.513	0.1422	0.0271	1.5×10^{-7}	0.0370	0.0269	0.169	0.328
rs11066008	12	112,140,669	G	A	0.351	0.1827	0.0301	1.2×10^{-9}	0.0102	0.0299	0.733	0.719
rs11066015[a]	12	112,168,009	A	G	0.275	0.1895	0.0292	8.1×10^{-11}	0.0066	0.0290	0.821	0.870
rs4646776	12	112,230,019	C	G	0.275	0.1906	0.0291	6.2×10^{-11}	0.0072	0.0290	0.805	0.876
rs671[a]	12	112,241,766	A	G	0.275	0.1894	0.0291	7.8×10^{-11}	0.0060	0.0290	0.836	0.878
rs78069066	12	112,337,924	A	G	0.280	0.1919	0.0295	7.7×10^{-11}	0.0060	0.0293	0.839	0.879
rs2339904	12	112,378,350	T	C	0.622	0.1437	0.0292	8.8×10^{-7}	0.0589	0.0291	0.043	0.198
rs11066132	12	112,468,206	T	C	0.271	0.1948	0.0303	1.2×10^{-10}	0.0006	0.0301	0.983	0.907
rs116873087	12	112,511,913	C	G	0.274	0.1904	0.0302	2.8×10^{-10}	-0.0024	0.0300	0.938	0.899
rs11066150	12	112,518,803	A	G	0.466	0.1739	0.0291	2.4×10^{-9}	0.0401	0.0290	0.167	0.464
rs147992802	12	112,552,274	T	C	0.344	0.1995	0.0305	6.3×10^{-11}	0.0257	0.0304	0.397	0.700
rs12231737	12	112,574,616	T	C	0.281	0.1939	0.0298	8.2×10^{-11}	0.0030	0.0297	0.918	0.903
rs144504271	12	112,627,350	A	G	0.277	0.1943	0.0297	5.8×10^{-11}	0.0036	0.0295	0.902	0.912
rs2074356[a]	12	112,645,401	A	G	0.252	0.2011	0.0299	1.8×10^{-11}	0.0000	0.0298	1.000	1.000
rs77768175	12	112,736,118	G	A	0.253	0.1974	0.0329	1.9×10^{-9}	-0.0083	0.0327	0.798	0.869
rs11066280[a]	12	112,817,783	A	T	0.290	0.1759	0.0286	8.0×10^{-10}	0.0018	0.0285	0.949	0.817
rs11537471	12	112,834,586	G	A	0.349	0.1852	0.0296	3.9×10^{-10}	0.0206	0.0294	0.485	0.678
rs139144808	12	113,470,025	TA	T	0.225	0.1638	0.0329	6.4×10^{-7}	0.0484	0.0327	0.140	0.298

[a] Directly genotyped; [b] Chromosome; [c] Chromosomal position (GRCh37/hg19); [d] Effect allele; [e] Non-effect allele; [f] Allele frequency of effect allele; [g] Effect size; [h] Standard error of effect size; [i] Linkage disequilibrium R^2 with rs2074356

Supplement Table 5. Results of genome-wide association study using both discovery and replication subjects

Adjustment	SNP	Chr[b]	Gene(s)	Position[c]	EA[d]	NEA[e]	Rsq[f]	AF[g]	Beta[h]	SE(Beta)[i]	Variance explained (%)	*P*
Age, Sex	rs573194563	5	*CT49–DNAH5 (intergenic)*	13,076,920	CA	C	0.810	0.955	0.2708	0.0539	0.31	5.0×10^{-7}
	**rs144504271**	**12**	***HECTD4 (intron)***	**112,627,350**	**A**	**G**	**0.955**	**0.265**	**0.1887**	**0.0237**	**0.67**	$\mathbf{1.5\times10^{-15}}$
Age, Sex, Smoking status	rs12094032	1	*MAB21L3–ATP1A1* (intergenic)	116,891,363	G	A	0.943	0.057	0.2139	0.0432	0.24	7.5×10^{-7}
	rs573194563	5	*CT49–DNAH5 (intergenic)*	13,076,920	CA	C	0.810	0.955	0.2605	0.0527	0.28	7.8×10^{-7}
	rs4410790[a]	7	*AGR3–AHR* (intergenic)	17,284,577	C	T	0.996	0.375	0.1020	0.0203	0.23	4.9×10^{-7}
	**rs2074356[a]**	**12**	***HECTD4 (intron)***	**112,645,401**	**A**	**G**	**0.996**	**0.240**	**0.1925**	**0.0234**	**0.65**	$\mathbf{1.7\times10^{-16}}$
Age, Sex, Smoking status, BM	rs12094032	1	*MAB21L3–ATP1A1* (intergenic)	116,891,363	G	A	0.943	0.057	0.2136	0.0432	0.24	7.8×10^{-7}
	rs573194563	5	*CT49–DNAH5 (intergenic)*	13,076,920	CA	C	0.810	0.955	0.2597	0.0527	0.28	8.4×10^{-7}
	rs4410790[a]	7	*AGR3–AHR* (intergenic)	17,284,577	C	T	0.996	0.375	0.1024	0.0203	0.24	4.4×10^{-7}
	**rs2074356[a]**	**12**	***HECTD4 (intron)***	**112,645,401**	**A**	**G**	**0.996**	**0.240**	**0.1920**	**0.0234**	**0.65**	$\mathbf{2.0\times10^{-16}}$

[a]Directly genotyped; [b]Chromosome; [c]Chromosomal position (GRCh37/hg19); [d]Effect allele; [e]Non-effect allele; [f]Imputation quality in terms of R-square calculated by the Minimac3 software version 1.0.11; [g]Allele frequency of effect allele; [h]Effect size; [i]Standard error of effect size

Results listed in bold are associations whose *P*-values were less than of genome-wide significance ($P < 5\times10^{-8}$).

Supplement Table 6. Minor allele frequency in the J-MICC samples for previously-reported SNPs

Locus	SNP	PubMed ID(s)	First Author	Year
2p24	rs1260326[a]	25288136	Cornelis MC	2015
4q22	rs1481012	25288136	Cornelis MC	2015
6q21	rs2216084	27561104	Pirastu N	2016
	rs6942255	27561104	Pirastu N	2016
	rs7745311	27561104	Pirastu N	2016
	rs7754744	27561104	Pirastu N	2016
	rs9386630[b]	27561104	Pirastu N	2016
7p21	rs4410790[a]	21490707; 25288136	Cornelis MC; Cornelis MC	2011; 2015
	rs6968554[a]	25288136	Cornelis MC	2015
	rs6968865[a]	21357676	Sulem P	2011
7q11.23	rs7800944	25288136	Cornelis MC	2015
	rs17685[a]	25288136	Cornelis MC	2015
7q31	rs382140[a]	21876539	Amin N	2012
11p13	rs6265[a]	25288136	Cornelis MC	2015
15q24	rs2470893[a]	21490707; 21876539; 25288136	Cornelis MC; Amin N; Cornelis MC	2011; 2012; 2015
	rs2472297[a]	21876539; 25288136; 21357676	Amin N; Cornelis MC; Sulem P	2012; 2015; 2011
	rs6495122[a]	21876539	Amin N	2012
17q11.2	rs9902453	25288136	Cornelis MC	2015

[a]These SNPs were directly Genotyped.

[b]These SNPs was not included in the reference panel (1000Genomes phase 3 version 5) and the genotype could not be imputed.

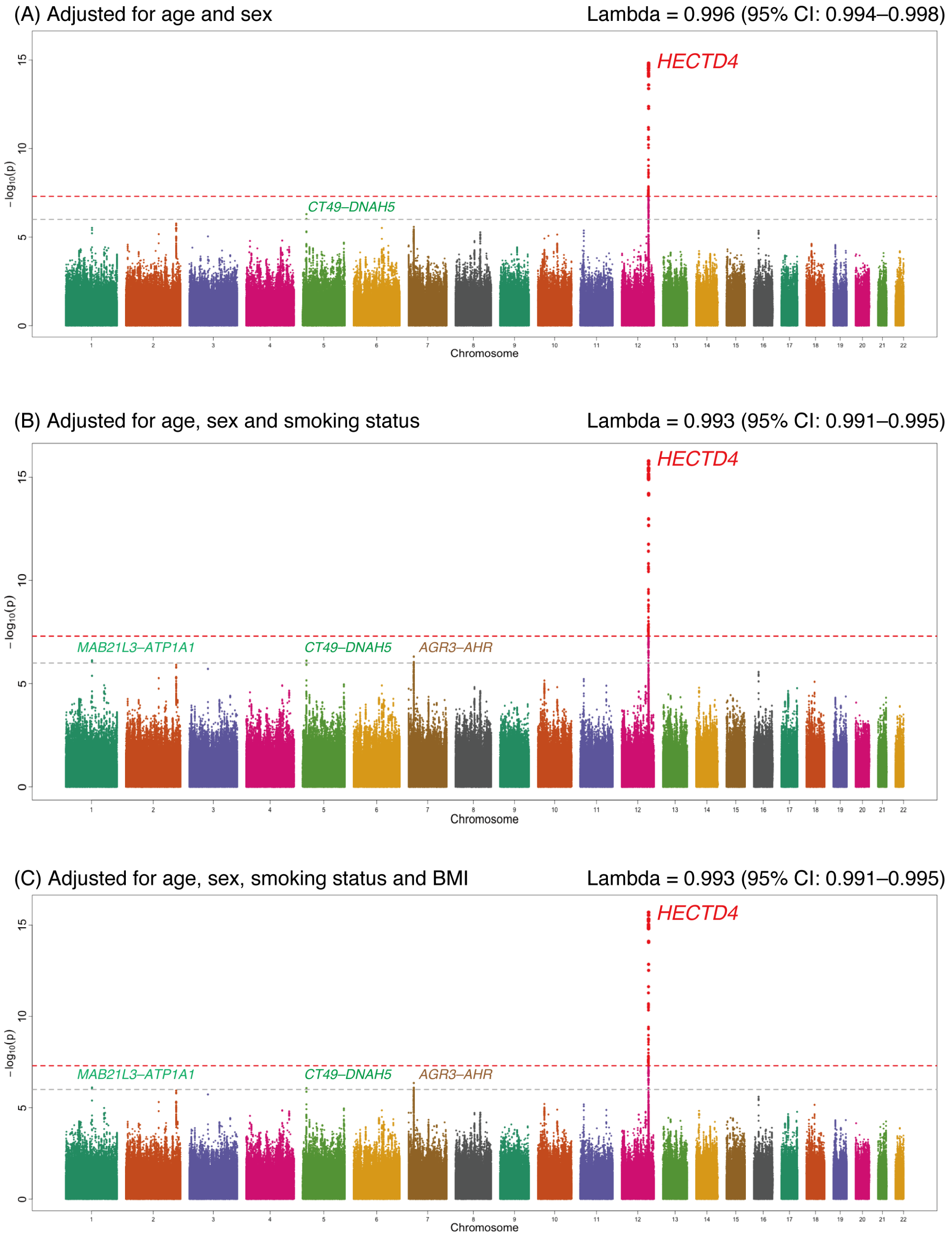

Figure S1. Genome-wide association signals from the combined analysis of discovery and replication samples (N = 11,261). The x-axis represents chromosomal positions and the y-axis represents $-\log_{10}$ P-values calculated by a mixed linear model association analysis. The grey and red dotted horizontal lines indicate the suggestive ($P = 1 \times 10^{-6}$) and genome-wide ($P = 5 \times 10^{-8}$) significance levels, respectively. Variants with

P-values indicating less than genome-wide significance (P <5 × 10^{-8}) are shown in red. Results were adjusted for age and sex (A); for age, sex, and smoking status (B); and for age, sex, smoking status, and BMI (C). The inflation factor, lambda, is the median of the observed test statistics divided by the median of the expected test statistics.

BMI, body-mass index; CI, confidence interval
